# Psychological impact of COVID-19 pandemic on postgraduate trainees: a cross-sectional survey

**DOI:** 10.1136/postgradmedj-2020-138364

**Published:** 2020-08-25

**Authors:** Nazish Imran, Hafiz Muhammad Umar Masood, Maryam Ayub, Khalid Masood Gondal

**Affiliations:** Child & Family Psychiatry Department, King Edward Medical University, Lahore, Pakistan; Fatima Memorial College of Medicine and Dentistry, Lahore, Pakistan; Academic Department of Psychiatry &behavioural Sciences, King Edward Medical University, Lahore, Pakistan; King Edward Medical University, Lahore, Pakistan

**Keywords:** Psychiatry, depression & mood disorders, adult psychiatry, education & training (see medical education & training), infectious diseases, medical education & training

## Abstract

**Background:**

The present study aimed to evaluate psychological impact of COVID-19 outbreak on postgraduate trainees in Pakistan by quantifying the symptoms of depression, anxiety and acute stress disorder and by analysing potential risk factors associated with these symptoms.

**Methods:**

Following Institutional Review Board approval, a cross-sectional study was conducted among 10,178 postgraduate trainees following COVID-19 outbreak through e-log system of College of Physicians and Surgeons of Pakistan. The nine-item Patient Health Questionnaire, seven-item Generalised Anxiety Disorder scale and Stanford Acute Stress Reaction Questionnaire were used to collect data. Statistical analyses were conducted using SPSS.26. Descriptive statistics, Mann-Whitney U test, the χ^2^ test and logistic regression analysis were performed. The significance level was set at α=0.05.

**Results:**

The prevalence of depressive symptoms, generalised anxiety disorder and acute stress disorder were 26.4%, 22.6% and 4.4%, respectively. Female postgraduate trainees, senior trainees and front-line workers reported experiencing more anxiety, depression and acute stress symptoms (p value<0.001). Logistic regression showed that being a front-line and senior staff member and female was associated with higher risk of experiencing symptoms of depression, anxiety and acute stress.

**Conclusions:**

Our study findings raise concerns about the psychological well-being of postgraduate trainees during the acute COVID-19 outbreak in Pakistan. It is necessary to employ strategies to minimise the psychological distress and provide adequate psychosocial support for postgraduate trainees during crisis situation such as COVID-19 pandemic.

## Introduction

The World Health Organization (WHO) identifies viral disease outbreaks like COVID-19 pandemic as a serious threat to public health.^1^ Similar to previous outbreaks like Severe Acute Respiratory Syndrome- Corona Virus (SARS-CoV) and Middle East respiratory syndrome, COVID-19 outbreak has also raised various difficulties for healthcare workers (HCWs) around the globe including increase workload, limited availability of personal protective equipment, scarcity of life-saving resource, frustration, isolation and ‘fear of possible infection in themselves and their families’.^2^ All these factors are likely to increase psychological distress among HCWs. Recent literature also suggests that HCWs are very vulnerable to emotional distress during COVID-19.^3–5^ Insufficient data are currently available from low- and middle-income countries (LMICs) regarding changes in prevalence of depression during the pandemic. However, a recent study conducted in India found 32.6% of HCWs having depression during COVID-19 pandemic, which is much higher than the 10% prevalence for common mental disorders reported in its general population.^6^

Postgraduate trainees are among the most vulnerable HCWs even in usual times with high prevalence of burnout and psychological morbidity.^7^ Previous studies report that junior doctors feel pressured and resentful that the primary care in acute situations is often left to them.^8^ During COVID-19 pandemic, postgraduate trainees are likely to be the main workforce to deal with the impending influx of patients with COVID-19. Mobilisation of postgraduate trainees to high demand departments, high likelihood of contacting suspected or confirmed cases with COVID-19, making difficult ethical decisions, cancellation of teaching programmes, and study leaves, uncertainty regarding jobs and rotations are some of the concerns identified by junior doctors during COVID-19 pandemic and are likely to adversely impact their mental and physical well-being.^9^ Similar views have been reported from Pakistan.^10^ A recent rapid review shows that presence on front lines, being younger and being more junior is associated with increased risk of psychological distress in HCWs during viral outbreaks.^2^ The psychological distress is associated with medical errors and lapses in professionalism as well as high risk of serious psychiatric problems including suicidal ideation.^11 12^

Although psychological distress is to be expected during COVID-19 in LMICs like Pakistan, where postgraduate trainees in particular are under pressure to look after a large number of potentially infectious patients, institutions, supervisors and employers can help to mitigate this by implementation of several effective interventions. However, their psychological well-being has not been formally evaluated so far. To address this gap, the present study aimed to evaluate psychological impact of COVID-19 outbreak on postgraduate trainees in Pakistan by quantifying the symptoms of depression, anxiety and acute stress, and by analysing potential risk factors associated with these symptoms.

## Methods

This was a cross-sectional study conducted following COVID-19 outbreak, through e-log system of College of Physicians and Surgeons (CPSP) from 15 April 2020 to May 2020. CPSP is the largest internationally recognised Postgraduate Medical Institute in Pakistan established in 1962 and is responsible for postgraduate medical training and research. It awards fellowships in 73 specialities/subspecialities and membership in 22 disciplines. All postgraduate trainees inducted from 2011 onwards in CPSP residency programmes have to make entries of all academic activities and work performed in mandatory e-logbook using their registration numbers and passwords. Trainees are unable to enter activities that are more than 3 months old. Supervisors verify the entries on a regular basis helping in monitoring the progress of trainees. Currently, there are 24, 477 postgraduate trainees enrolled with CPSP. Our study target population was postgraduate trainees who would access their e-logbook during the study duration from 15 April 2020 to May 2020. All trainees accessing e-portal were able to see the survey and answer the questionnaire by clicking on the relevant link. Among the 11, 489 trainees, who accessed e-log portal during the study period, 10, 178 participated with a response rate of 88.6%. Institutional Review Board approved the study. Participation was voluntary; all study participants provided informed consent, and confidentiality of information was assured. Demographic data collected from the participants included age, gender, marital status, speciality, year of residency, and medical and psychiatric history. Residents who were directly involved in diagnosing, treating or providing care to patients with diagnosed or suspected patients with COVID-19 on self-report were classified as front-line workers, while others were considered as second-line workers. Furthermore, postgraduate trainees in the first 2 years of residency were classified as junior trainees and those in year 3 and year 4 were considered as senior residents. The questionnaire included three validated questionnaires in English language focusing on depression, anxiety and acute stress disorder. The nine-item Patient Health Questionnaire (PHQ-9) was used to screen and measure the severity of symptoms of depression.^13^ It has nine items, score of each item ranges from 0 to 3 with total score range being 0–27. The scores are interpreted as normal (0–4), mild (5–9), moderate (10–14) and severe (15–21) depression. As recommended, PHQ-9-total score of 8 points or greater was defined as the presence of depressive symptoms for the current study.^14^ The seven-item Generalised Anxiety Disorder (GAD-7; range 0–21) was used to assess the severity of symptoms of anxiety.^15^ The scores are interpreted as normal (0–4), mild (5–9), moderate (10–14) and severe (15–21) anxiety. Stanford Acute Stress Reaction Questionnaire (SASRQ; range 0–150) questionnaire was used to measure residents acute stress in accordance with Diagnostic and Statistical Manual of Mental Disorders, fourth edition, criteria for acute stress disorder.^16^ The SASRQ is a six-point Likert scale consisting of 30 items assessing dissociation, re-experiencing, avoidance, anxiety and hyperarousal. Each item has a score of between 0 and 5, with a combined score ranging from 0 to 150, and a higher score indicating higher levels of self-reported stress. To meet acute stress disorder criteria, respondent had to indicate having at least three of five possible dissociation symptoms, and at least one symptom each of hyperarousal, anxiety, avoidance and re-experiencing of stressful event. Information to access confidential psychological support was also provided during the study. Data were analysed using SPSS version 26.0 (IBM Corp., NY, USA). A descriptive analysis was conducted using numbers and percentages for categorical data and mean±SD for continuous data. Medians with IQRs were reported for the skewed data values, Mann-Whitney U test was performed for comparison between the groups. Data for each level for symptoms of depression, anxiety, and stress were presented as numbers and percentages. The significance level was set at α=0.05. Qualitative variables were compared using χ^2^ test. Logistic regression analysis was performed to observe the potential risk factors for symptoms of depression, anxiety and acute stress in participants. Factors with significant association (p<0.05) with the outcome variables at univariate analysis level were further included in the final multivariate analysis model. The associations between risk factors and outcomes are presented as ORs and 95% CIs, after adjustment for confounder variables.

## Results

A total of 10, 178 postgraduate residents completed the survey with majority (56%) being female and belonging to surgery and allied speciality (55%). A total of 3767 (37%) were directly involved in diagnosing, treating or providing care to patients with diagnosed or suspected patients with COVID-19 (front-line workers). Table 1 gives further demographic details of the study participants (table 1).

**Table 1:** Demographic characteristics of respondents (N=10 178)

Characteristics	N (%)
Age, mean (SD)	31.50 (6.9)
Gender	
Women	5776 (56.7)
Men	4402 (43.3)
Marital status	
Single	7051 (69.3)
Married	3127 (30.7)
Speciality	
Medicine and allied	4222 (41.4)
Surgery and allied	5628 (55.2)
Basic sciences	328 (3.22)
Residency year	
PG1	3071 (30.2)
PG2	3610 (35.5)
PG3	2265 (22.3)
PG4	1232 (12.1)
Province	
Punjab	4867 (47.8)
Sindh	2469 (24.3)
Khyber Pukhtunkhwa	2369 (23.3)
Balochistan	328 (3.2)
Azad Kashmir	145 (1.4)
Working position	
Front-line	3767 (37.0)
Second-line	6411 (63.0)
H/O medical illness	
Yes	1150 (11.3)
No	9028 (88.7)
H/O psychiatric illness	
Yes	338 (3.3)
No	9840 (96.7)

The prevalence of depressive symptoms, GAD and acute stress disorder was 26.4%, 22.6% and 4.4%, respectively (table 2). The values for women were higher than those for man in all scales (p value<0.001). The median (IQR) scores on the PHQ-9 for depression, the GAD-7 for anxiety and the SASRQ for acute stress disorder for all respondents were 4.0 (1.0–9.0), 3.0 (0.0–7.0) and 11.0 (2.0–28.0), respectively. Female residents, front-line workers and senior residents had higher scores in depression, anxiety and acute stress subscales compared with those men, second-line workers and junior staff (table 3, online supplemental table S1). All these three groups also reported experiencing more severe symptom levels on psychiatric outcome variables (table 4).

**Table 2:** Prevalence of depression, anxiety and acute stress disorder among residents during COVID-19 outbreak stratified by gender

Variable	Total (N=10 178) n(%)	Males (N=4402) n(%)	Females (N=5776) n(%)	P value
Depression (PHQ-9)*				
Absent	7493 (73.6)	3400 (77.2)	4093 (70.9)	<0.001
Present	2685 (26.4)	1002 (22.8)	1683 (29.1)	
Generalised anxiety disorder (GAD)†				
Absent	7877 (77.4)	3551 (80.7)	4326 (74.9)	<0.001
Present	2301 (22.6)	851 (19.3)	1450 (25.1)	
Acute stress disorder (SASRQ)				
Absent	9727 (95.6)	4241 (96.3)	5486 (95.0)	<0.001
Present	448 (4.4)	161 (3.7)	287 (5.0)	

* Depressive symptoms included individuals who scored ≥8 points.

† GAD was defined as individuals who scored ≥7 points.

PHQ-9, nine-item Patient Health Questionnaire; SASRQ, Stanford Acute Stress Reaction Questionnaire.

**Table 3:** Scores of depression, anxiety and Stanford Acute Stress Questionnaire and its subscales during COVID-19 outbreak in total cohort and subgroups

		Gender			Working position			Seniority		
		Median (IQR)			Median (IQR)			Median (IQR)		
Scale	Total score Median (IQR)	Men	Women	P value	Front-line	Second-line	P value	Senior resident	Junior resident	P value
PHQ-9, depression symptoms	4.0 (1.0–9.0)	3.0 (0–8.0)	5.0 (1.0–9.0)	<0.001	5.0 (1.0–10.0)	3.0 (0–8.0)	<0.001	4.0 (1.0–9.0)	4.0 (1.0–9.0)	<0.05
GAD-7, anxiety symptoms	3.0 (0–7.0)	2.0 (0–6.0)	4.0 (0–8.0)	<0.001	4.0 (0–8.0)	3.0 (0–7.0)	<0.001	3.0 (0–7.0)	3.0 (0–7.0)	<0.05
SASRQ (acute stress symptoms)	11.0 (2.0–28.0)	10.0 (1.0–25.0)	13.0 (3.0–30.0)	<0.001	14.0 (4.0–30.0)	10.0 (2.0–26.0)	<0.001	12.0 (2.0–30.0)	11.0 (2.0–27.0)	0.124
Dissociation subscale	2.0 (0–8.0)	2.0 (0–7.0)	3.0 (0–9.0)	<0.001	3.0 (0–9.0)	2.0 (0–7.0)	<0.001	3.0 (0–9.0)	2.0 (0–8.0)	<0.05
Re-experiencing subscale	1.0 (0–5.0)	1.0 (0–5.0)	2.0 (0–5.0)	<0.001	2.0 (0–6.0)	1.0 (0–5.0)	<0.001	1.0 (0–5.0)	1.0 (0–5.0)	<0.05
Avoidance subscale	3.0 (0–6.0)	2.0 (0–6.0)	3.0 (0–7.0)	<0.001	3.0 (0–7.0)	3.0 (0–6.0)	<0.001	3.0 (0–6.0)	3.0 (0–6.0)	0.758
Anxiety subscale	2.0 (0–6.0)	2.0 (0–5.0)	3.0 (0–6.0)	<0.001	3.0 (0–7.0)	2.0 (0–6.0)	<0.001	3.0 (0–6.0)	2.0 (0–6.0)	<0.05

GAD-7, seven-item Generalised Anxiety Disorder; PHQ-9, nine-item Patient Health Questionnaire; SASRQ, Stanford Acute Stress Reaction Questionnaire.

**Table 4:** Severity categories of depression, anxiety and acute stress disorder in total cohort and stratified by gender, working position and seniority

		Gender			Working position			Seniority		
		N (%)			N (%)			N (%)		
Severity category	Total No (%) 10 178	Men	Women	P value	Front-line	Second-line		Junior resident	Senior resident	P value
PHQ-9, depression symptoms										
Normal	5482 (53.9)	2605 (59.2)	2877 (49.8)	<0.001	1820 (48.3)	3662 57.1)	<0.001	3670 (54.9)	1812 (51.8)	<0.001
Mild	2475 (24.3)	979 (22.2)	1496 (25.9)		998 (26.5)	1477 (23.0)		1611 (24.1)	864 (24.7)	
Moderate	1254 (12.3)	476 (10.8)	778 (13.5)		521 (13.8)	733 (11.4)		813 (12.2)	441 (12.6)	
Severe	967 (9.5)	342 (7.8)	625 (10.8)		428 (11.4)	539 (8.4)		587 (8.8)	380 (10.9)	
GAD-7, anxiety symptoms										
Normal	6055 (59.5)	2896 (65.8)	3159 (54.7)	<0.001	2049 (54.4)	4006 (62.5)	<0.001	4034 (60.4)	2021 (57.8)	0.06
Mild	2421 (23.8)	887 (20.1)	1534 (26.6)		960 (25.5)	1461 (22.8)		1560 (23.3)	861 (24.6)	
Moderate	1089 (10.7)	438 (10.0)	651 (11.3)		473 (12.6)	616 (9.6)		704 (10.5)	385 (11.0)	
Severe	613 (6.0)	181 (4.1)	432 (7.5)		285 (7.6)	328 (5.1)		383 (5.1)	230 (6.6)	
SASRQ, acute stress disorder										
No	9727 (95.6)	4241 (96.3)	5486 (95.0)	<0.001	3562 (94.6)	6165 (96.2)	<0.001	6417 (96.1)	3310 (94.7)	<0.001
Yes	448 (4.4)	161 (3.7)	287 (5.0)		205 (5.4)	243 (3.8)		262 (3.9)	186 (5.3)	

GAD-7, seven-item Generalised Anxiety Disorder; PHQ-9, nine-item Patient Health Questionnaire; SASRQ, Stanford Acute Stress Reaction Questionnaire.

The associations of potential risk factors with depressive symptoms, GAD and acute stress disorder during COVID-19 outbreak are presented in table 5. In the logistic regression models, being female (OR=1.48, 95% CI 1.35 to 1.63) and front-line worker during COVID-19 (OR=1.35, 95% CI 1.23 to 1.48) and senior resident (OR=1.2, 95% CI 1.09 to 1.32) were significantly associated with depression in residents. Similarly, being female, senior resident and front-line worker during COVID-19 also appeared to be an independent risk factor for anxiety and acute stress symptoms after adjustment (table 5).

**Table 5:** Risk factors for mental health outcomes identified by multivariate logistic regression analysis

Variable	Adjusted OR (95% CI)	P value
PHQ-9, depression symptoms		
Gender		
Men	(Reference)	<0.001
Women	1.48 (1.35 to 1.63)	
Seniority		
Junior resident	(Reference)	<0.001
Senior resident	1.20 (1.09 to 1.32)	
Working position		
Second-line	(Reference)	<0.001
Front-line	1.35 (1.23 to 1.48)	
GAD-7, anxiety symptoms		
Gender		
Men	(Reference)	<0.001
Women	1.5 (1.35 to 1.65)	
Seniority		
Junior resident	(Reference)	<0.05
Senior resident	1.12 (1.01 to 1.24)	
Working position		
Second-line	(Reference)	<0.001
Front-line	1.45 (1.32 to 1.6)	
SASRQ, acute stress disorder		
Gender		
Men	(Reference)	<0.001
Women	1.35 (1.1 to 1.66)	
Seniority		
Junior resident	(Reference)	<0.001
Senior resident	1.36 (1.12 to 1.65)	
Working position		
Second-line	(Reference)	<0.001
Front-line	1.44 (1.19 to 1.75)	

GAD-7, seven-item Generalised Anxiety Disorder; PHQ-9, nine-item Patient Health Questionnaire; SASRQ, Stanford Acute Stress Reaction Questionnaire.

## Discussion

COVID-19 pandemic had a considerable impact on healthcare systems worldwide, and threaten not only the physical health but also psychological and social health of HCWs including junior doctors. To our knowledge, this study is the first large-scale national survey in Pakistan to investigate the psychological impact of COVID-19 pandemic on postgraduate trainees. This cross-sectional survey enrolled 10, 178 respondents, and overall, our findings raise concerns about the psychological well-being of postgraduate trainees involved in the acute COVID-19 outbreak.

Even prior to pandemic, LMICs have reported higher prevalence of depressive disorders and anxiety than the aggregate prevalence for very high human development index countries in the world.^17 18^ People in LMICs, including Pakistan, are exposed to more stressors, which alongside greater stigma and social inequalities leads to less likelihood of accessing timely treatment. In the absence of any large population-based studies from Pakistan looking at the relative change or increase in anxiety and depression during the COVID-19 pandemic, we are unable to comment that symptoms observed in postgraduate trainees in our study differ from those of the general population.

Our study contributes to literature on prevalence of psychological symptoms in postgraduate trainees. Prevalence of GAD and depression as 22.6% and 26.4%, respectively, in our sample are in line with few recent studies of HCWs during COVID-19. Zhu *et al* reported prevalence of anxiety symptoms as 24.1%, while in another web-based survey of HCWs in China, 19.8% of HCWs appeared to have depressive symptoms.^4 19^ However, these results are in contrast with extremely high prevalence (35%–50%) of psychological morbidity in some reports from China, particularly in Wuhan province and in neighbouring India.^3 4 6^ Similarly, prevalence of acute stress disorder as 4.4% in our sample, although closer to 5% observed in a study following SARS outbreak in Taiwan in 2004, is very low compared to 29.8% reported by Zhu *et al* during current COVID-19 outbreak.^19 20^ This comparatively low psychological morbidity in our sample may partially be explained due to relatively lower (2.1%) COVID-19 case fatality of Pakistan as compared to USA (5.9%), Italy (14.3%), UK (14.0%), Iran (5.3%) and China (5.5%).^21^ Another reason may be the timings of the study, as it was conducted prior to the peak of the CoV outbreak in Pakistan, when healthcare system was coping well comparatively. The differences in the results between various studies in the literature can also be contributed to variability in study settings, methodologies, instruments used to assess the psychological morbidity and participants’ backgrounds such as age and culture.

Previous small-scale studies done to assess psychological morbidity among postgraduate trainees in Pakistan found that 14% had depression symptoms and 8% had moderate anxiety.^22 23^ Thus, although many other factors may have contributed, our results indicate that COVID-19 outbreak perhaps led to increase in prevalence of depression and anxiety among postgraduate trainees in Pakistan. Increase workload, sleep deprivation, being junior resident and pay disparity were observed to be associated with depression in junior doctors in Pakistan in literature.^22^ These stressors are likely to be compounded by high risk of infection, inadequate safety equipment, social isolation— especially from family—and physical exhaustion leading to high psychological morbidity during these challenging times.^10^ Female postgraduate trainees in our sample had higher prevalence of psychological morbidity as well as more severe symptoms on all psychological measures. Previous studies have also demonstrated that female gender is associated with increased vulnerability to psychological distress.^2 3 24^ Similar to recent literature, front-line workers and those directly exposed to patients with COVID-19 in our sample had high risk of developing psychological symptoms.^2 3 24^ This is understandable given that not only front-line postgraduate trainees are more exposed to the risk of COVID-19 infection themselves but also have to deal with the sickest patients.

Our results of senior postgraduate trainees having significantly high psychological morbidity than year 1 and year 2 trainees are different from existing literature, in which less clinical experience has been linked with high adverse psychological outcome.^2 3^ This could be explained by observed practice in hospitals in Pakistan of senior postgraduate trainees being given more responsibility of looking after COVID-19 units, intensive care units and high dependency units, thus perhaps being more exposed to patients at greatest risk of dying from the COVID-19 illness. However, this finding warrants further research.

Although all the health resources of the country are currently deployed towards service provision for increasing number of patients with COVID-19, the policy makers in teaching and training institutions need to make special efforts to promote the psychological well-being of post-graduate trainees. Self-care, need to maintain healthy balance lifestyle during residency training and in future careers and building skills in resilience should be emphasised in the training curriculum. Adequate training around infectious diseases and provision of adequate personal protective equipment should be recommended to all institutions. The postgraduate trainees should have appropriate work shifts, regular breaks and guaranteed supplies. Encouragement among peers, adequate supervision and access to psychological interventions should be guaranteed in order to deal with the psychological problems.^25^ Psychological first aid for front-line workers has also been recommended by WHO and includes the assessment of needs and concerns; practical care and support; basic needs provision; empathic listening; and access to information, services and social supports. These steps can allow postgraduate trainees to function at their best during this global health emergency.

The study has some limitations. Although all scales showed very good reliability (Cronbach’s alpha >0.9) in our sample, only PHQ-9 and GAD-7 has been previously validated in Pakistani population. As the study was cross sectional, we cannot evaluate the temporality and causality of the observed factors. Psychological assessment in our study was based on self-report tools. The use of clinical interviews may help in a more comprehensive assessment. We also cannot exclude the possibility of a response bias. Almost 47% of total enrolled residents in e-log portal (11 489/24 477) accessed it during the study period. It may be possible that many medical residents, who were directly participating in the care of patients with COVID-19 and thus having increase likelihood of psychological morbidity, were too busy to log in during the study duration. Also, residents who saw but did not responded to survey may have been too stressed out and overwhelmed to respond, thus skewing the results. Alternatively, those who received the survey but did not reply may have had no distress and therefore were not interested in responding. Furthermore, our study was conducted during a critical period of the COVID-19 pandemic in Pakistan when cases were still rising but prior to mid-June 2020 (the days that Pakistan saw the peak of COVID-19 cases and most likely high stressful time for HCWs). Longitudinal approach might help verifying whether long-term overload and distress develops as cases with COVID-19 reach their peak in the country and whether psychiatric disorders, especially posttraumatic stress disorder, might occur with the COVID-19 progression.

Despite the limitations, this study has significant strengths. To the best of our knowledge, this is the first national study to report the psychological symptoms among postgraduate trainees during the COVID-19 pandemic. The data represented all four provinces and included trainees from multiple specialities. Thus, the results of the study can be considered representative of postgraduate trainee doctors’ psychological well-being during this outbreak.

To conclude, the present study provides insight into the potential immediate psychological sequelae of COVID-19 pandemic on postgraduate trainees in the resource-constrained setting of a LMIC. Our results show high levels of depression and anxiety experienced by trainees caring for patients with COVID-19 in Pakistan. Female residents, those in the third and fourth year of residency and front-line workers experience more psychological distress. It is necessary to employ strategies to minimise the psychological distress and provide adequate psychosocial support for postgraduate trainees during a crisis situation such as COVID-19. Further research is needed to assess the long-term impact of this outbreak on trainee mental health as well as effectiveness of interventions to improve their psychological well-being.

**Main messages**

- High psychological distress among postgraduate trainees during COVID-19 pandemic is reported in Pakistan.
- Female postgraduate trainees and those working as front-line healthcare workers reported experiencing more anxiety, depression and acute stress symptoms.
- Senior postgraduate trainees reported more anxiety and depression symptoms.
- Provision of adequate psychosocial support for postgraduate trainees during a crisis situation such as COVID-19 outbreak is essential.

**Current research questions**

- Studies outside Pakistan, with larger sample size and similar/uniform scales and cut-off points are needed for comparison.
- Further research is needed to explore the reasons for senior residents having high prevalence of anxiety and depression.
- Longitudinal studies with larger sample sizes are needed to understand the long-term mental health consequences of this devastating global pandemic on postgraduate trainees.
- Qualitative research will also be helpful to gain insight into the impact of COVID-19 pandemic on postgraduate trainees’ psychological well-being.

**What is already known on the subject**

- The unprecedented nature of the COVID-19 pandemic challenges is likely to cause extreme psychological stress among the healthcare workers.
- Front-line healthcare workers, women, junior staff and nurses are more likely to suffer anxiety and depression.

## Supplementary Material

postgradmedj-97-632-DC1-inline-supplementary-material-1 Table S1: Mean Scores & Standard deviation of Depression, Anxiety & Stanford Acute Stress Questionnaire and its subscales during COVID-19 outbreak in total cohort and subgroups.
