# Supplementary material for: Psychological impact of COVID-19 pandemic on postgraduate trainees: a cross-sectional survey
Source: Postgrad Med J. 2020 Aug 25;97(1152):632–7. doi: 10.1136/postgradmedj-2020-138364 (PMC7447959; doi:10.1136/postgradmedj-2020-138364)
Supplement: postgradmedj-97-632-DC1-inline-supplementary-material-1 — Table S1: Mean Scores & Standard deviation of Depression, Anxiety & Stanford Acute Stress Questionnaire and its subscales during COVID-19 outbreak in total cohort and subgroups. [file postgradmedj-97-632-dc1-inline-supplementary-material-1.pdf]

Psychological Impact Of COVID-19 Pandemic On Postgraduate Trainees: A Cross Sectional Survey

SUPPLEMENTAL FILE

Table S1 : Mean Scores & Standard deviation of Depression, Anxiety & Stanford Acute Stress Questionnaire and its subscales during COVID-19 outbreak in total cohort and subgroups.

		Gender			Working Position			Seniority		
		Mean (SD)			Mean (SD)			Mean (SD)		
**Scale**	**Total Score Mean (SD)**	**Men**	**Women**	**P. value**	**Frontline**	**Second line**	**P. value**	**Senior Resident**	**Junior Resident**	**P. value**
**PHQ-9 Depression symptoms**	5.65(5.92)	4.94(5.56)	6.20(6.12)	<0.001	6.36(6.13)	5.24(5.75)	<0.001	5.93(6.14)	5.51(5.80)	<0.001
**GAD-7, anxiety symptoms**	4.63(5.16)	3.92(4.80)	5.17(5.36)	<0.001	5.26(5.39)	4.26(4.99)	<0.001	4.83(5.30)	4.53(5.09)	<0.05.
**SASRQ (Acute stress symptoms)**	20.30 (25.42)	18.03 (23.43)	22.03 (26.72)	<0.001	22.85 (27.12)	18.80 (24.25)	<0.001	21.58 (27.13)	19.63 (24.46)	<0.001
**Dissociation subscale**	5.70(8.25)	4.98(7.52)	6.25(8.73)	<0.001	6.47(8.79)	5.24(7.89)	<0.001	6.19(8.84)	5,44(7.92)	<0.001
**Re-experiencing subscale**	3.52(5.23)	3.15(4.81)	3.81(5.52)	<0.001	4.08(5.65)	3.20(4.94)	<0.001	3.79(5.58)	3.39(5.04)	<0.001
**Avoidance subscale**	4.74(5.86)	4.24(5.43)	5.12(6.14)	<0.001	5.15(6.48)	4.49(5.65)	<0.001	4.90(6.12)	4.65(5.72)	<0.05.
**Anxiety subscale**	4.43(5.62)	3.80(5.16)	4.91(5.90)	<0.001	5.04(5.96)	4.07(5.37)	<0.001	4.73(5.94)	4.27(5.43)	<0.001

Abbreviations: PHQ-9, The 9-item Patient Health Questionnaire: GAD-7, 7-item Generalized Anxiety Disorder: SASRQ, Stanford Acute stress Reaction Questionnaire) :SD, Standard deviation
